# Measurement Disparities in Frailty Among Kidney Transplant Patients: Impact of Differential Item Functioning

**DOI:** 10.1093/geroni/igab046.1728

**Published:** 2021-12-17

**Authors:** Nadia Chu, Alden Gross, Xiaomeng Chen, Qian-Li Xue, Karen Bandeen-Roche, Dorry Segev, Mara McAdams-DeMarco

**Affiliations:** 1 Johns Hopkins University School of Medicine, Baltimore, Maryland, United States; 2 Johns Hopkins Bloomberg School of Public Health, Baltimore, Maryland, United States

## Abstract

Frailty is commonly measured for clinical risk stratification during transplant evaluation and is more prevalent among older, non-White kidney transplant (KT) patients. However, group differences may be partially attributable to misclassification resulting from measurement bias (differential item functioning/DIF). We examined the extent that DIF affects estimates of age, sex, and race differences in frailty (physical frailty phenotype/PFP) prevalence among 4,300 candidates and 1,396 recipients. We used Multiple Indicators Multiple Causes with dichotomous indicators to assess uniform DIF in PFP criteria attributable to age (≥65vs.18-64 years), sex, and race (Black vs.White). Among candidates (mean age=55 years), 41% were female, 46% were Black, and 19% were frail. After controlling for mean frailty level, females were more likely to endorse exhaustion (OR=1.20,p=0.003), but less likely to endorse low activity (OR=0.83,p=0.01). Younger candidates were more likely to endorse weight loss (OR=1.30,p=0.005), exhaustion (OR=1.60,p<0.001), and low activity (OR=1.80,p<0.001). Black candidates were more likely to endorse exhaustion (OR=1.25,p<0.001), but less likely to endorse weakness (OR=0.79,p<0.001). Among recipients (mean age=54 years), 40% were female, 39% were Black, and 15% were frail. Younger recipients were more likely to endorse weight loss (OR=1.55,p=0.005) and low activity (OR=1.61,p=0.02); however, no DIF was detected by sex or race. Results highlight the impact of DIF for specific PFP measures by age, sex, and race among candidates, but only by age for recipients. Further research is needed to ascertain whether candidate- and/or recipient-specific thresholds to correct for DIF could improve risk prediction and equitable access to KT for older, female, and Black candidates.

